# Quantum crystallography applied to RNA in Olex2

**DOI:** 10.1063/4.0000873

**Published:** 2025-10-27

**Authors:** Blaine H. M. Mooers

**Affiliations:** University of Oklahoma Health Sciences Center, Oklahoma City, OK, USA

## Abstract

The use of scattering factors for nonspherical atoms in structure refinement generally requires subatomic resolution diffraction data. The promises of this approach are a dramatic reduction in the R-factor and higher-quality electron density maps that can help further resolve disorder in structure. This resolution requirement limits the application of this methodology to biological molecules because so few give X-ray diffraction data with such high diffraction limits.

We have been working with a well-characterized RNA quadruplex to explore new methods of optimizing crystal growth. Crystals of these RNAs sometimes diffract to subatomic resolution in the hands of others (Deng et al. 2001; Fyfe et al. 2015). We re-explored crystallization space and found that this RNA can crystallize in new space groups. Some of our crystals gave data to 0.67 Angstroms, allowing nonspherical atomic scattering factors to be used via NoSpherA2 in Olex2 (Kleemiss et al. 2021).

The quantum chemistry software used by NoSpherA2 is not designed to handle biological polymers. We mapped each RNA atom to a unique identifier, like in chemical crystallography. We then translated the unique atom names back to their standard names as members of a polynucleotide chain after refinement of the scattering factors using quantum mechanics. We compare the results of applying NoSpherA2 in Olex2, multipole refinement with MoPro, and conventional refinements with anisotropic atomic displacement parameters in ShelxL and Phenix refinements.
